# Discoidin domain receptors; an ancient family of collagen receptors has major roles in bone development, regeneration and metabolism

**DOI:** 10.3389/fdmed.2023.1181817

**Published:** 2023-05-11

**Authors:** Renny T. Franceschi, Shawn A. Hallett, Chunxi Ge

**Affiliations:** Department of Periodontics and Oral Medicine, University of Michigan School of Dentistry, Ann Arbor, MI, United States

**Keywords:** extracellular matrix, collagen receptor, differentiation, stem cell, bone, cartilage

## Abstract

The extracellular matrix (ECM) niche plays a critical role in determining cellular behavior during bone development including the differentiation and lineage allocation of skeletal progenitor cells to chondrocytes, osteoblasts, or marrow adipocytes. As the major ECM component in mineralized tissues, collagen has instructive as well as structural roles during bone development and is required for bone cell differentiation. Cells sense their extracellular environment using specific cell surface receptors. For many years, specific β1 integrins were considered the main collagen receptors in bone, but, more recently, the important role of a second, more primordial collagen receptor family, the discoidin domain receptors, has become apparent. This review will specifically focus on the roles of discoidin domain receptors in mineralized tissue development as well as related functions in abnormal bone formation, regeneration and metabolism.

## 1. Introduction to collagen receptors

As the most abundant class of ECM proteins, collagens provide structural support for connective tissues, skin and, most importantly, bones and teeth, and can convey information about the extracellular mechanical environment via their interaction with cells using specific collagen receptors. The importance of collagen to bone development is well established; collagen synthesis is necessary for differentiation of skeletal progenitors to osteoblasts (1–4) and conditions that interfere with collagen synthesis or structure *in vivo* such as vitamin C deficiency or osteogenesis imperfecta severely disrupt bone development (5–8).

Until recently, it was generally assumed that bone cells interacted with the collagenous ECM exclusively through integrins, the best-known ECM receptors. Through their linkage with the cytoskeleton, integrins are major force transducers linking the ECM microenvironment with cellular functions including nuclear transcription (9). The collagen-binding integrins all have a common β1 subunit and four different alpha subunits to produce α1β1, α2β1, α10β1 and α11β1 integrins, which are all detected in bone (10–13). Disruption of integrin-collagen binding in cell culture using blocking antibodies to specific integrin subunits inhibits osteoblast differentiation of skeletal progenitor cells including preosteoblast cell lines and primary bone marrow cell cultures (12, 14–16). Because of their shared β1 subunit, the overall requirement for collagen-binding integrins in bone was assessed *in vivo* using conditional inactivation of the β1 integrin gene (*Itgb1*). Using this approach, bone phenotypes of varying severity were observed with the strongest effects of *Itgb1* inactivation being associated with expression of Cre recombinase early in the bone lineage and milder phenotypes seen at later stages. For example, *Itgb1* inactivation in embryonic mesenchymal progenitors using *Twist2-Cre* was associated with severe bone phenotypes and perinatal lethality (17). Disruption at later stages using *Osx-Cre* (preosteoblast stage) reduced skeletal growth, mineralization and mechanical properties, effects that became progressively milder with age while disruption of *Itgb1* with *Bglap-Cre* had only minor effects on skeletal development (17, 18). Similarly, *Itgb1* inactivation in cartilage using *Col2a1-Cre* resulted in perinatal lethality in most pups, stunted cartilage growth and disruption of chondrocyte proliferation and polarity (19). Although in some cases loss of *Itgb1* function severely retarded bone development, in no case was bone formation and mineralization completely disrupted. This shows that some degree of bone formation can occur in the absence of collagen-binding integrins and suggests the involvement of other collagen receptors.

Interestingly, the collagen-binding integrins appeared relatively late in evolutionary history, being first seen with the emergence of chordates (20). In contrast, collagen-like proteins are present in all metazoan species (21). The discoidin domain receptors (DDRs) are a more ancient class of cell-surface collagen binding proteins than integrins. Like collagens, they are present in most invertebrate metazoans including *Caenorhabditis elegans*, *Drosophila melanogaster*, and *Hydra vulgaris* and so could function as collagen receptors before the collagen-binding integrins appeared on the scene. Although functions of DDRs in invertebrates have not been extensively examined, in *C. elegans*, specific DDR functions have been described related to axonal guidance which also requires collagen. Since DDRs have likely functioned as collagen receptors over a much longer period of time than integrins, they may have more primordial functions related to collagen signaling [for review, see reference (22)].

As will be discussed, DDRs are very different from integrins in terms of their interaction with collagens, structure, mechanism of action, tissue distribution and activity in specific cell populations. This review will specifically focus on roles of DDRs in mineralized tissues. However, it should be noted that DDRs also have non-skeletal functions in epithelial and connective tissues and have been linked to several diseases including cancer, fibrosis, and kidney disease that will not be discussed here. The reader is referred to several excellent reviews for a comprehensive treatment of these diverse DDR activities (23–26).

## 2. DDR structure and function

**Figure 1:**
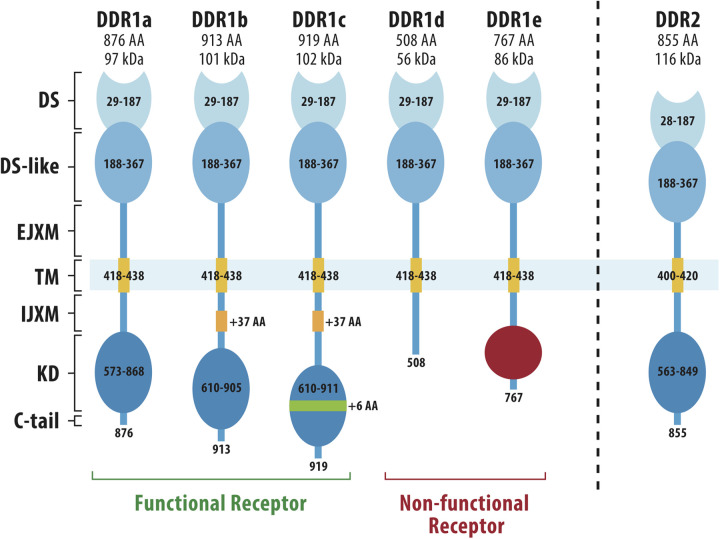
Structures of DDR1 and DDR2. DDR1 has 5 different spliced variants (DDR1a-e) while DDR2 exists only as a single protein. N-terminal DS (discoidin) and DS-like (discoidin-like) globular domains are shared by all DDR1 spliced variants and share high homology with the same domains in DDR2. Other regions are an extracellular juxtamembrane domain (EJXM), a transmembrane domain (TM), an intracellular juxtamembrane domain (IJXM), a kinase domain (KD) and a short C-terminal tail. The collagen-binding pocket is contained within the DS domain. Adapted from Rammal et al. (30).

Unlike integrins, which lack intrinsic kinase activity, the DDRs are collagen-activated receptor tyrosine kinases (RTKs) that share homology in their kinase domain with growth factor receptors such as the neurotrophin receptor, TrkA (25, 27, 28). DDRs are named for their homology to the *Dictyostelium discoideum* lectin, discoidin. In mammals, there are two DDR proteins, DDR1 and DDR2, which show different preferences for binding to fibrillar and non-fibrillar collagens. Both DDR1 and 2 bind type I, II, III and V fibrillar collagens. In contrast, DDR1 selectively binds basement membrane type IV collagen while DDR2 binds type X collagen (27–29). The overall structural features of DDR1 and 2 are summarized in Figure 1. Starting from the N-terminus, both proteins have an extracellular DS domain, the region of homology with discoidin, a DS-like domain, a juxtamembrane domain, a single pass transmembrane domain, an intracellular juxtamembrane domain and an intracellular kinase domain. DS and DS-like domains and the kinase domain are highly conserved between DDR1 and DDR2. The DS domain distinguishes the DDRs from other RTKs and contains the binding site for triple-helical collagens (31, 32). DDR1 exists in 5 different spliced forms while only a single DDR2 protein has been described. In DDR1, the extracellular and transmembrane domains are shared between all 5 isoforms while there are several differences in the cytoplasmic domains. Two of the 5 DDR1 splice variants lack a functional kinase domain and could potentially act as decoy receptors for the kinase-containing isoforms (25).

Like the collagen-binding integrins, the DDRs only bind to native triple-helical collagens [i.e., thermally denatured collagen cannot serve as a binding substrate (21, 28, 31)]. DDR1 and 2 both bind a 6 amino acid sequence present in fibrillar collagens I-III, GVMGFO, where O is hydroxyproline (33, 34). This same sequence is also recognized by two other collagen-binding proteins, Secreted Protein Acidic and Rich in Cysteine (SPARC) and von Willebrand Factor that have functions in collagen mineralization and the blood coagulation cascade, respectively (35, 36). The GVMGFO sequence is distinct from the motif recognized by collagen-binding integrins which has the consensus sequence, GxOGEx (e.g., GFOGER or GAOGER in fibrillar collagens) (37, 38). Interestingly, in the COL1A1, COL2A1 and COL3A1 chains of types I–III collagen, the O of GVMGFO and the G of GFOGER/GAOGER are separated by 96 amino acid residues, a finding with possible implications concerning coupling between DDRs and integrins (see Section 6). The interaction between the DDR2 DS domain and a triple-helical peptide containing the GVMGFO sequence has been examined at atomic resolution using x-ray crystallography (39). These studies identified an amphiphilic binding pocket for the GVMGFO sequence that is conserved between DDR2 and DDR1. One side of this pocket contains apolar amino acid residues (Trp52, Thr56, Asn175, Cys73-Cys177) while the other side contains polar residues forming a salt bridge (Arg105-Glu113, Asp69) (39).

Like other RTKs, the DDRs are ligand-activated tyrosine kinases. However, instead of responding to soluble molecules such as growth factors, the DDRs have high molecular weight triple-helical collagen as a ligand. They differ from classic RTKs in other ways as well. Instead of existing as monomers that dimerize with ligand binding, DDRs are homodimers in the unactivated state (40, 41). Also, instead of being activated by their ligands and undergoing autophosphorylation within seconds to minutes like other RTKs, DDR phosphorylation takes hours and can often persist for days after binding collagen (27, 28). No truly satisfactory explanation for this phenomenon has been advanced although the involvement of secondary cellular processes such as oligomerization or internalization may be important (40, 42). Since DDRs are activated with similar kinetics by small triple-helical peptides containing the GVMGFO core binding sequence, higher order fibrillar structure of native collagen is not required for this unusual behavior (33, 34).

Once activated, DDRs stimulate several downstream signals including ERK1/2 and p38 mitogen-activated protein kinase, phosphatidylinositol-3-kinase/AKT and NF-Kβ pathways. DDRs may also have functions separate from their kinase activities, possibly related to the control of collagen fibrillogenesis and/or orientation (43, 44). It is not the purpose of this review to provide a comprehensive discussion of DDR2 signaling mechanisms as these have been thoroughly reviewed by others [see ref (23, 25)].

## 3. Tissue distribution of DDR1 and DDR2 in mineralized tissues

Initial evaluation of *Ddr1* and *Ddr2* mRNA distribution suggested that *Ddr1* is predominantly expressed in epithelial tissues, smooth muscle and immune cells while *Ddr2* is in connective tissues (45). More recently, tissue distribution was assessed by immunohistochemistry and *in situ* hybridization as well as by using a LacZ knock-in *Ddr2* mutant where a bacterial β-galactosidase gene was inserted into the *Ddr2* locus. The following discussion will emphasize DDR distribution in mineralized tissues.

### 3.1. DDR1

Although an early study that measured DDR1 binding sites in mice using DDR1 extracellular domain fused with alkaline phosphatase showed binding to all skeletal structures, skin and the urogenital tract because of their high collagen content (46), studies that actually measured the tissue distribution of DDR1 protein or mRNA are quite limited. In neonatal and adult mice, DDR1 was localized by immunohistochemistry to proliferating and hypertrophic chondrocytes of long bone growth plates, cortical and trabecular bone osteocytes, periosteum, and articular chondrocytes (47–49). *In situ* hybridization analysis was conducted in oral tissues using a *Ddr1* probe (50). Consistent with an epithelial pattern of expression, highest *Ddr1* mRNA levels were detected in oral epithelium including enamel organs of developing molars and basal cell layers of the oral epithelium, but low expression in ectomesenchymal tissues.

### 3.2. DDR2

Early *in situ* hybridization studies localized *Ddr2* expression to tibial growth plates (51). Subsequent more detailed analysis using *Ddr2^+/LacZ^* mice stained for β-galactosidase activity, first detected *Ddr2* expression in bone rudiments at E11.5 (52, 53). Analysis from E13.5 through adulthood showed strong staining in all developing skeletal elements in the appendicular, axial and cranial skeletons including growth plate cartilage, metaphyses, periosteum, cranial sutures and cranial base synchondroses. In general, expression was higher in cells representing earlier stages of each skeletal lineage. For example, in growth plates and synchondroses, expression was higher in resting and proliferating zone cells and lower in hypertrophic layers. Also, while *Ddr2* was detected in marrow and periosteal/preosteoblast layers near forming trabecular and cortical bone surfaces, no expression was detected in osteocytes. Similar periosteal localization was reported using immunohistochemistry where DDR2 colocalized with alkaline phosphatase, a preosteoblast marker (54). Notably, this distribution is very different from most of the collagen-binding integrins (α1β1, α2β1, α11β1) that are broadly expressed in connective tissues [reviewed in ref (55)]. However, there may be some overlap with integrin α10β1 which shows preferential expression in chondrocytes (11, 56). *Ddr2^+/LacZ^* mice were also used to examine *Ddr2* expression during tooth development (57) and in the temporomandibular joint (TMJ) (58). *Ddr2* was widely expressed in non-epithelial tooth structures including dental follicle and dental papilla during development and odontoblasts, alveolar bone osteoblast and periodontal ligament fibroblasts of adults. In contrast to the *Ddr1* mRNA distribution described above, it was conspicuously absent from epithelial structures including ameloblasts and Hertwig's epithelial root sheath. Strong *Ddr2* expression was also detected in the TMJ articular surface of adult mice. Interestingly, at this age, *Ddr2* expression in the articular surface of the knee joint was quite low suggesting differences between the fibrocartilage of the TMJ and hyaline cartilage of the knee (58).

### 3.3. Localization of DDR2 in skeletal progenitor cells

To gain further insight into the lineage of *Ddr2*-expressing cells, *Ddr2^mer−icre−mer^*; *ROSA26^LSLtdTomato^* mice were developed (52, 53). After tamoxifen-induced recombination, *Ddr2*-expressing cells are labelled with tdTomato fluorescent protein, thereby allowing these cells to be followed over time. Mice were injected with tamoxifen from P1-P4 and *tdTomato+* cells were lineage-traced for up to 2 months. Initially, *tdTomato+*cells had a similar distribution to that seen in *Ddr2^+/LacZ^* mice with labelling in growth plate and synchondrosis resting zone, cranial sutures, perichondrium, trabeculae, and periosteum, but absent in more differentiated cells. Over time, *tdTomato*^+^ cells appeared in proliferating and hypertrophic chondrocytes, osteoblasts and, eventually, osteocytes. Osteoclasts were not labelled. This result is what would be expected if *Ddr2* was expressed in skeletal progenitor cells (SPCs) whose progeny became the mature cells of each skeletal lineage (hypertrophic chondrocytes for the cartilage lineage, osteocytes for the osteoblast lineage). Consistent with this concept, a high degree of colocalization between DDR2 and the skeletal progentitor/stem cell marker, GLI1 (59, 60), was observed by immunofluorescence in cranial sutures, synchondroses and tibial growth plates (52, 53). Also, CD140α^+^/CD51^+^ SPCs purified from bone marrow by FACS were enriched in *Ddr2* mRNA (52).

Further evidence for DDR2 being a marker for skeletal stem cells comes from a recent study published in preprint form where DDR2 was detected in a unique cranial suture cell population (61) that could be distinguished from previously described CTSK+ suture stem cells (SSCs) (62). These DDR2^+^ cells have several stem cell properties including long cycling time, capacity for self-renewal after *in vivo* implantation, potential to differentiate to osteoblasts, adipocytes and chondrocytes, expression of several SC markers including GLI1 and capacity to generate all DDR2+ cells present in the native suture. Interestingly, conditional ablation of *Ctsk*-labeled SSCs using diphtheria toxin administration to *iDTR*; *Ctsk-Cre* mice led to increased expansion of DDR2^+^ suture cells and suture fusion via an endochondral mechanism. The authors postulate that DDR2^+^ suture stem cells contribute to a novel form of endochondral ossification without hematopoietic recruitment, a third potential mechanism of bone formation.

### 3.4. Regulation of *Ddr2* transcription

The transcriptional control mechanisms regulating DDR2 levels in bone cells are not well understood. To date they have only been examined in cell culture where *Ddr2* is upregulated during osteoblast differentiation (63–65). One possible factor controlling this upregulation is ATF4 which, together with C/EBPβ, interacts with a C/EBP binding site at −1,150 bp in the *Ddr2* promoter to stimulate *Ddr2* expression and subsequent increases in osteoblast marker mRNAS (65). However, it is not known if these control mechanisms function *in vivo* or if other factors participate in this regulation.

## 4. Genetic models for understanding DDR functions in mineralized tissues

Experiments of nature (i.e., human genetic diseases) as well as gene inactivation mouse models have been described that, taken together, provide considerable insight into how DDRs function in bone, cartilage and the dentition.

### 4.1. Human loss-of-function mutations in *DDR2* are associated with severe skeletal and craniofacial defects while gain-of-function mutations cause fibrosis and skull abnormalities

To date, no human mutations in *DDR1* have been identified. In contrast, genetic disorders have been described associated with both loss and gain-of-function mutations in *DDR2*. Spondylo-meta-epiphyseal dysplasia with short limbs and abnormal calcifications (SMED, SL-AC) is a rare autosomal recessive genetic disorder first described in 1993 that is associated with dwarfism, short limbs, reduced bone mass, abnormal skull shape including mid-face hypoplasia and hypertelorism, open fontanelles, micrognathia and tooth abnormalities (66). This disorder was subsequently mapped to chromosome 1q23, the locus of *DDR2*, and shown to be caused by loss-of-function mutations in the DDR2 tyrosine kinase domain as well as mutations affecting intracellular trafficking (67–70). Unfortunately, individuals with this disorder rarely survive beyond childhood; atlantoaxial instability and resulting spinal cord damage is the most common cause of death (71, 72). The short lifespan of SMED, SL-AC patients compounded with the rarety of this disorder have limited studies in humans.

A second disorder, designated as Warburg-Cinotti Syndrome, was described in 2018 and associated with putative activating mutations in the DDR2 kinase domain (73). Fibroblasts from patients exhibited high levels of DDR2 phosphorylation in the absence of collagen stimulation, suggesting that receptor activation was ligand-independent. This disorder, which is inherited in an autosomal dominant manner, is associated with progressive fibrosis, corneal vascularization, skull abnormalities and osteolysis. In view of the deleterious effects of *DDR2* loss-of-function mutations on bone formation in SMED, SL-AC pateints, it is not clear why activating mutations would lead to an osteolytic phenotype. However, since only 6 patients with Warburg-Cinotti Syndrome have been described, the phenotypic variation within this disorder cannot be currently assessed.

*DDR2* may also be a determinant of bone mineral density (BMD) and fracture risk in human populations. Analysis of a Chinese Han population and an American Caucasian population identified 28 SNPs in *DDR2*. Of these, 3 were significantly associated with hip BMD in the Chinese, but not in the American population (74). Although this preliminary finding suggests that certain polymorphisms in *DDR2* may be risk factors for osteoporosis, more studies are needed, particularly in diverse populations to assess the significance of these findings.

As will be described below, the phenotypic similarities between SMED, SL-AC patients and *Ddr2*-deficient mice indicate that mouse models are an appropriate model for studying this disease.

### 4.2. Global *Ddr1* and *Ddr2* knockout models suggest roles in bone and tooth development

As shown in early studies, global knockout of either *Ddr1* or *Ddr2* resulted in dwarf phenotypes, particularly for *Ddr2*-deficient mice (46, 51). However, different bases for the observed growth deficits were proposed. In *Ddr1* deficient mice, all organs were proportionally smaller suggesting an overall growth defect (46). However, no differences in growth plate size, chondrocyte proliferation or apoptosis were noted.

In contrast, initial analysis of globally *Ddr2* deficient mice showed prominent growth retardation that was attributed to decreased proliferation of growth plate chondrocytes in the absence of changes in apoptosis resulting in shortened growth plates (51). A similar phenotype was subsequently observed in *Ddr2^slie/slie^* mice, which have a spontaneous 150 kb deletion in *Ddr2* that encompasses exons 1–17 to produce an effective null allele (75). A more detailed analysis of the bone phenotype of *Ddr2^slie/slie^* mice revealed that skeletal growth defects were accompanied by large reductions in trabecular bone volume, trabecular thickness and number, changes that were attributed to reduced bone formation rate rather than stimulation of osteoclastic bone resorption (65). Similar changes in vertebral trabecular bone were also seen. However, cortical bone was only slightly affected. Interestingly, the reduction in bone mass with *Ddr2* deficiency was accompanied by an increase in marrow fat. Consistent with these changes, bone marrow stromal cells (BMSCs) or calvarial preosteoblasts cultured from *Ddr2^slie/slie^* mice exhibited defective osteoblast differentiation while differentiation of BMSCs to adipocytes was enhanced.

Changes in craniofacial morphology in *Ddr1* and *Ddr2*-deficient mice have been compared using a machine learning approach that was able to clearly discriminate between skulls from wildtype, *Ddr1* and *Ddr2*-deficient mice (76). Although *Ddr1*-deficient skulls are somewhat smaller than wild type controls, they have no substantial alterations in relative skull dimensions. In contrast, skulls from *Ddr2*-deficient mice are dramatically shorter in the anterior-posterior direction with a more spherical skull shape associated with increased anterior skull width as well as reduced nasal bone length. Subsequent analysis of this phenotype identified a defect in proliferation of synchondrosis chondrocytes, particularly in the intersphenoid synchondrosis, in the absence of changes in apoptosis (53). These changes were associated with a characteristic expansion of the synchondrosis resting zone, possibly related to the defective conversion of these cells into proliferating chondrocytes. *Ddr2*-deficient skulls also have open fontanelles at birth, thinning of frontal bones and defects in frontal suture fusion that persist into adulthood (53, 65).

Effects of global *Ddr1* and *Ddr2* inactivation on the dentition were also examined. *Ddr1*-deficient mice had normal teeth, but age-dependent periodontal degeneration including alveolar bone loss was noted (50). In contrast, teeth from *Ddr2^slie/slie^* mice had smaller roots and reduced crown/root ratio resulting in disproportionate tooth size (57). These mice also exhibited gradual alveolar bone loss over a 10-month period due to increased osteoclast activity as well as atypical periodontal ligament collagen fibrils.

### 4.3. Conditional *Ddr1* and *Ddr2* inactivation studies in bone

In addition to affecting the skeleton, global *Ddr1* deficiency inhibits uterine development and embryo implantation as well as mammary epithelium development leading to defective milk production (46). Likewise, *Ddr2* deficiency reduces fertility by inhibiting female and male gonadal function and steroid hormone production leading to partial sterility and interferes with certain metabolic activities (75) (see Section 8). Because effects of global inactivation of *Ddr1* or *Ddr2* are not restricted to the skeleton, specific cell-autonomous functions of these collagen receptors in bone cannot be inferred from global knockout studies. Although several early studies with osteoblast and chondrocyte cell lines and primary cultures suggested direct functions for DDR1 and 2 in bone cells (48, 63, 64), this issue was not resolved until recently when results of tissue-specific *Ddr1* and *Ddr2* knockouts were reported.

#### 4.3.1. Ddr1

Chondrocyte or osteoblast-selective inactivation of *Ddr1* was achieved by crossing *Ddr1^fl/fl^* mice with *Col2a1^CreERT^* or *Col1a1^CreERT^* mice (47–49). Chondrocyte-selective knockout of *Ddr1* in tamoxifen-treated *Col2a1^CreERT^*;*Ddr1^fl/fl^* mice led to a 10–20 percent decrease in body weight and length and delayed formation of a secondary ossification center (47). In contrast to early reports with global *Ddr1* knockouts (46), decreases in chondrocyte proliferation, apoptosis and hypertrophy were reported (47). These changes were accompanied by an approximately 20 percent change in trabecular bone volume while cortical thickness was unchanged. In addition, the chondrocyte hypertrophy markers (ColX, MMP13, RUNX2) and hedgehog pathway intermediate, IHH, all decreased. These results suggest that inactivation of *Ddr1* in chondrocytes preferentially affects endochondral ossification. Results with *Col1a1^CreERT^*;*Ddr1^fl/fl^* mice, where *Ddr1* was preferentially inactivated in osteoblasts/osteocytes were markedly different from chondrocyte-selective knockouts (48). In this case, minimal changes in endochondral ossification or trabecular bone parameters were noted while cortical thickness was reduced by approximately 50 percent. These changes were accompanied by a loss of mechanical properties and inhibition of osteoblast markers such as RUNX2, ALPL, BGLAP and COLIA1. In a second study with *Col1a1^CreERT^*;*Ddr1^fl/fl^* mice, the same group examined the consequences of *Ddr1* inactivation in adults over extended periods (49). In this case, modest changes in trabecular parameters were noted together with reductions in cortical thickness, osteoblast differentiation markers and cortical bone formation rate. These changes were accompanied by increased apoptosis and autophagy markers. No craniofacial changes were described in any of these studies.

#### 4.3.2. Ddr2

Conditional knockout studies with *Ddr2* were informed by results of localization and lineage tracing experiments showing preferential expression of this gene in GLI1+ skeletal progenitor cells, chondrocytes, and osteoblasts (see Sections 3.2, 3.3). To determine functions of *Ddr2* in these cells, *Ddr2^fl/fl^* mice were crossed with Gli*1^CreERT^*, *Col2a1^Cre^*or *Bglap^Cre^* mice and resulting long bone and craniofacial phenotypes were examined (52, 53). Inactivation of *Ddr2* in *Gli1*-expressing cells, induced by injecting neonatal *Gli1^CreERT^*;*Ddr2^fl/fl^* mice with tamoxifen, resulted in essentially the same phenotype seen in *Ddr2^slie/slie^* mice. Dwarfism was observed in both males and females, and this was associated with an approximately 12 percent reduction of growth plate length at P14. In addition, severe defects in endochondral bone formation were observed, particularly in males where trabecular BV/TV was reduced by approximately 50 percent. Associated reductions in trabecular number and thickness and increased trabecular spacing were also seen at 3 months. However, cortical BV/TV was not affected. The craniofacial phenotype of *Gli1^CreERT^*;*Ddr2^fl/fl^* mice was also essentially identical to *Ddr2^slie/slie^* mice; anterior-posterior skull length was reduced with an associated increase in anterior skull width. Mutants also exhibited frontal bone thinning and shortened nasal bones (53). Also like global knockouts, the anterior portion of frontal sutures failed to mineralize in most mice.

The phenotype of *Col2a1^Cre^*;*Ddr2^fl/fl^* mice was similar to *Gli1^CreERT^*;*Ddr2^fl/fl^* and *Ddr2^slie/slie^* mice with the important exception that no defects in suture fusion were observed. Although it has been proposed that changes in growth of the cranial base can affect suture fusion (77), this is clearly not an adequate explanation for effects of *Ddr2* inactivation on frontal sutures since *Col2a1^Cre^*;*Ddr2^fl/fl^* mice had the same cranial base growth defects seen in *Gli1^CreERT^*;*Ddr2^fl/fl^* mice. Based on this result, it was concluded that functions of *Ddr2* in synchondrosis endochondral bone formation are independent from its functions in cranial sutures. Consistent with the observed reduction in tibial bone formation, mRNA levels of osteoblast and hypertrophic chondrocyte markers were all reduced in *Col2a1^Cre^*;*Ddr2^fl/fl^* mice. These changes were accompanied by decreased mRNA levels of the hedgehog pathway intermediates, *Ihh* and *Gli1*. Since defects in Hh signaling were also noted with conditional *Ddr1* knockout (47) (Section 4.3.1), this pathway may be a common target for DDRs.

Although *Ddr2* was expressed in osteoblasts on trabecular and periosteal surfaces, it probably does not have a major function in mature osteoblasts since *Bglap^Cre^*;*Ddr2^fl/fl^* mice were essentially identical to wild type control mice. Because this Cre is mainly active in mature osteoblasts and, possibly, osteocytes, it is still possible that *Ddr2* may have functions in earlier stages of the osteoblast lineage.

Overall, *Ddr2* conditional knockout studies support the concept that this gene functions in earlier stages of bone formation (i.e., in *Gli1^CreERT^*-positive skeletal progenitor cells and *Col2a1^Cre^*-positive resting zone and proliferative chondrocytes) rather than in terminally differentiated osteoblasts or hypertrophic chondrocytes. Two cell culture studies reinforce this conclusion (52). In the first, E12.5 limb buds from *Ddr2^fl/fl^* mice were used to prepare micromass cultures enriched in chondro-osteo progenitors that were treated with control or Cre adenovirus before growth in chondrogenic medium. *Ddr2* inactivation strongly inhibited chondrogenesis as measured by Alcian blue staining or expression of chondrocyte markers. In the second study, CD140α^+^/CD51^+^ SPCs were prepared from *Ddr2^fl/fl^* mice and grown in osteogenic medium after treatment with Cre adenovirus. In this case, *Ddr2* inactivation strongly inhibited osteoblast differentiation (mineralization and expression of osteoblast markers).

#### 4.3.3. Possible functions of *Ddr2* in osteoclasts

The studies described above all focused on functions of *Ddr2* in chondro-osteo lineage cells which form chondrocytes, osteoblasts and osteocytes. However, there is still some controversy regarding possible *Ddr2* functions in osteoclasts. On one hand, lineage tracing studies with *Ddr2^mer−icre−mer^*; *ROSA26^LSLtdTomato^* mice did not show colocalization of the tdTomato label with TRAP-positive osteoclasts (Section 3.3) and globally *Ddr2* deficient mice (*Ddr2^slie/slie^* mice) did not have any detectable changes in bone resorption markers or osteoclast differentiation capacity (Section 4.1). On the other hand, evidence was presented that DDR2 has a suppressive effect on osteoclast formation in cell culture models (78). DDR2 protein and mRNA were detected at low levels in the RAW264.7 macrophage cell line and primary cultures of bone marrow macrophage and these levels were further reduced with *in vitro* induction of osteoclast formation. Also, overexpression of *Ddr2* in RAW264.7 was shown to inhibit osteoclast induction while shRNA knockdown of *Ddr2* further stimulated this process. Furthermore, adenovirus-mediated overexpression of *Ddr2* in the femur marrow cavity partially reversed osteoporosis in ovariectomized mice, a phenotype that is largely due to osteoclast activation. These studies suggest that *Ddr2* can function in the monocytic lineage to suppress osteoclastogenesis. Lastly, in a recent study *Ddr2^fl/fl^* mice were crossed with *LysM^Cre^* mice to conditionally inactivate *Ddr2* in myeloid lineage cells (79). The resulting animals had a hyperinflammatory phenotype after exposure to either collagen antibody-induced arthritis or a high-fat diet. After arthritis induction, mice had increased ankle inflammation, elevation of inflammatory markers, increased bone resorption and increased osteoclast surface per bone surface as well as an approximately 15 percent decrease in bone mineral density. Also, evidence was presented that loss of DDR2 increased macrophage repolarization from an M2 to M1 phenotype resulting in enhanced inflammation. However, this study did not look for changes in bone density in the absence of an inflammatory stimulus. Nevertheless, this work supports a role for DDR2 in the suppression of osteoclastogenesis through its inhibitory actions on monocytic osteoclast precursors. However, it is still not clear why, in previous studies, changes in bone resorption markers were not detected in *Ddr2^slie/slie^* mice or why osteoclasts were not detected as part of the DDR2 lineage (52, 65). It is possible that effects on bone resorption in the absence of induced inflammation may not be large enough to affect bone mass or, alternatively, that in globally *Ddr2* deficient mice, interference with other DDR2 dependent processes may compensate for effects on osteoclastogenesis. Another possibility would be that DDR2 is not expressed in the osteoclast lineage and does not have a direct function in these cells, but rather modulates effects of macrophage on osteoclastogenesis. Studies where *Ddr2* is more selectively inactivated only in osteoclasts (for example, using *Ctsk-Cre* or *TRAP-Cre*) may be necessary to resolve this issue (80).

### 4.4. DDR2-dependent changes in osteoblast gene expression

A consistent finding from *Ddr2* knockout studies is that osteoblast differentiation and associated expression of osteoblast marker genes are suppressed. A limited number of studies have investigated the basis for this suppression. Because of its central role as a master transcriptional regulator of bone formation, studies to date have focused on RUNX2. This transcription factor is expressed at early times during bone development coincident with the formation of cartilage condensations and has roles in both hypertrophic cartilage formation as well as osteoblast differentiation [for review (81)]. RUNX2 activity is subject to several controls including phosphorylation by ERK1/2 and p38 mitogen-activated protein kinases (MAPKs) (82). Both MAPKs are important for bone formation as demonstrated by *in vivo* gain and loss-of-function studies (83, 84). Once activated, MAPKs translocate to the nucleus where they bind and phosphorylate RUNX2 on the chromatin of target genes (85). MAPKs phosphorylate RUNX2 on several serine residues, the most important being Ser301 and Ser319 (86). Phosphorylated RUNX2 recruits specific histone acetyltransferases and methylases to chromatin resulting in increased H3K9 and H4K5 acetylation and H3K4 di-methylation, histone modifications associated with transcriptional activation, as well as decreased H3K9 mono-, di and tri-methylation, histone marks associated with repression (85). These changes open chromatin structure to allow RNA polymerase II to bind and initiate transcription of osteoblast-related genes. RUNX2 phosphorylation and MAPK activity are obligatory for these changes since transfection of cells with a phosphorylation-resistant S301,319A mutant RUNX2 or treatment with MAPK inhibitors blocks transcription.

Since both ERK1/2 and p38 MAPKs are known downstream responses to DDR2 activation (25), it was hypothesized that this pathway could explain the observed stimulatory effects of DDR2 on osteoblast gene expression. This concept has been tested in cell culture studies with osteoblast cell lines as well as in osteoblasts from *Ddr2*-deficient mice (64, 65). In early studies with osteoblast cell lines and primary BMSC cultures, DDR2 was shown to stimulate osteoblast differentiation through a pathway involving ERK/MAPK activation and RUNX2 phosphorylation (64). *Ddr2* shRNA inhibited differentiation while overexpression was stimulatory. These changes were paralleled by increased or decreased ERK/MAPK activity, RUNX2 phosphorylation and transcriptional activity. Significantly, effects of *Ddr2* shRNA knockdown could be overcome by transfecting cells with a phosphomimetic Runx2 S301,319E mutant where replacement of alanine with glutamate mimics a phosphate group. In separate studies referenced in Section 4.1 (65), calvarial preosteoblasts or BMSCs isolated from *Ddr2^slie/slie^* mice were found to be deficient in ability to undergo osteoblast differentiation while BMSCs from these mice exhibited enhanced adipogenic differentiation. The reduced osteoblast differentiation in *Ddr2*-deficient cells was directly related to reduced ERK/MAPK activity and RUNX2-S319 phosphorylation and was rescued by transfection with the RUNX2 S301/319E mutant described above. The ability of DDR2 to stimulate ERK/MAPK activity may also explain the increase in marrow fat observed in *Ddr2^slie/slie^* mice. In addition to phosphorylating RUNX2, ERK1/2 can phosphorylate the adipogenic transcription factor, PPARγ, on Ser112. In this case, however, phosphorylation inhibits transcriptional activity. By preventing this inhibitory phosphorylation, *Ddr2* knockout would be expected to restore PPARγ activity to permit formation of marrow fat. Consistent with this interpretation, transgenic mice containing a phosphorylation-resistant S112A PPARγ mutant have increased marrow fat and reduced bone mass (87).

## 5. Requirement for DDR2 in bone regeneration

Consistent with the marked effects of *Ddr2* deficiency on bone development, inactivation of this gene was also shown to inhibit bone regeneration. Two regeneration models were examined, a calvarial bone defect and a tibial fracture (88, 89). For the calvarial model, a 0.5 mm burr hole defect was generated in wild type or *Ddr2^slie/slie^* mice and regeneration was examined for increasing times up to 12 weeks. In wild type mice, this type of defect was completely healed after 4 weeks while no bone bridging was seen in mutant mice even after 12 weeks. *Ddr2*, which was expressed in sutures and periosteal cells before injury, was detected in the injury site within 3 days and expanded during the healing process. Also, inactivation of *Ddr2* in calvarial cells in culture reduced osteoblast differentiation. For the fracture model, a mid-shaft tibial fracture was created in wild type or *Ddr2^slie/slie^* mice and fracture healing was monitored for 3 weeks. In this case, *Ddr2-*deficient mice were unable to form complete unions at the fracture site as measured by Radiographic Union Score Tibia (mRUST) (90).

## 6. Functions of DDR2 in cartilage matrix organization and relationship to ECM stiffness

In the studies described above, the reduced linear growth of long bones and skulls in *Ddr2*-deficient mice was attributed to proliferation defects in growth plate and synchondrosis chondrocytes in the absence of changes in apoptosis (52, 53). Interestingly, an examination of chondrocyte morphology revealed that the normal organization of these cells into columns was disrupted with *Ddr2* inactivation. This effect was seen in long bone growth plates but was particularly striking in cranial base synchondroses where the central resting zone was greatly expanded with widely separated disorganized cells (52, 53). In some cases, chondrocytes actually shifted their orientation by 90 degrees to form an ectopic hypertrophic zone at right angles to the normal plane of synchondrosis organization. These changes were accompanied by loss of chondrocyte polarity as measured by disruption of the normally consistent orientation of GM130, a Golgi apparatus marker, relative to the nucleus and anterior-posterior axis of the skull. This may explain the proliferation defect seen in chondrocytes of *Ddr2*-deficient mice since disruption of GM130 orientation is known to impair spindle assembly and cell division (65). The relevance of these findings to human physiology is emphasized by the observation that collagen matrix distribution is also disrupted in growth plate cartilage from SMED, SL-AC patients (66).

How might DDR2 affect chondrocyte polarity? One possibility is that it is necessary for collagen matrix organization and fibril orientation which would subsequently affect chondrocyte orientation. Examination of the type II collagen distribution in both growth plates and synchondroses by immunofluorescence microscopy revealed a shift from a uniform distribution in the territorial matrix next to chondrocytes and the extraterritorial matrix between cell clusters in wild type mice to an uneven distribution restricted to the pericellular space adjacent to chondrocytes in mutants (53). These changes were accompanied by loss of type II collagen fibril orientation as measured by second harmonic generation (SHG) microscopy. This analysis detected a dramatic shift from a highly oriented matrix (high anisotrophy) in synchondroses of wild type mice to a disorganized matrix (low anisotropy) in mutants where fibrils had a randomized orientation (53). Although primary cilia have been related to cell polarity and collagen orientation in other systems (91), regulation of this important organelle by DDR2 has not been reported.

Another consequence of DDR2 maintaining collagen fibril orientation is an increase in overall ECM stiffness. Although this has not been examined during bone development, there are several examples in other experimental systems. For example, DDR2 in breast cancer-associated fibroblasts (CAFs) increases tumor stiffness by organizing type I collagen fibrils (92). Also, at sites of trauma-induced heterotopic ossification, DDR2 increases collagen fibril orientation as measured by SHG (93) (also see Section 7.3). In both cases, evidence was presented that DDR2 functioned in concert with collagen-binding β1 integrins to stimulate, on one hand, tumor metastasis to the lungs or, on the other, ectopic bone formation. As noted in Section 2, fibrillar collagens I–III containing binding sites for both DDRs and integrins are always separated by 96 amino acid residues. This characteristic spacing may allow collagen to simultaneously regulate both these receptors. For example, in breast tumor metastasis, DDR2 was found to stimulate CAF-mediated mechanotransduction by increasing integrin activation in response to collagen. This was accomplished by stimulating RAP1-mediated Talin1 and Kindlin2 recruitment to integrins in focal adhesions (92). Also, in trauma-induced heterotopic ossification, DDR2 was necessary for full activation of integrin-dependent signals such as focal adhesion kinase (FAK) activation as well as nuclear levels of the Hippo pathway intermediate, TAZ, and its downstream targets (93).

## 7. Involvement of DDRs in abnormal ossification

Given the involvement of DDRs in normal bone formation, it is not totally surprising that they are also involved when this process goes awry. It this section, DDR involvement in vascular calcification, osteoarthritis and heterotopic ossification will be discussed.

### 7.1. Vascular calcification

Initiated by insults such as high levels of circulating LDL cholesterol, diabetes or chronic kidney disease, vascular calcification is a key event in advanced atherosclerosis. Calcium phosphate crystals can be deposited either in the subepithelial intima of blood vessels (intimal calcification) or in the smooth muscle-rich media (medial calcification) (94). This latter process shares many similarities with normal bone formation. It is initiated by differentiation of vascular smooth muscle cells or SMC progenitors into osteochondroprogenitor cells which form bone-like structures in arteries through a process that mimics endochondral bone formation as indicated by formation of cartilage that subsequently is converted into a bone-like structure (95). Like normal bone formation, this process requires interactions of progenitor cells with type I collagen and is mediated by the master transcriptional regulator of bone formation, RUNX2 (96, 97). Vascular calcification can be induced in mice by feeding LDL receptor-deficient animals (*Ldlr^−/−^* mice) a high fat, high cholesterol diet. Breeding a *Ddr1*-null allele into *Ldlr^−/−^* mice resulted in animals that were resistant to developing vascular calcification (97). Subsequent analysis showed that calcification was inhibited via a mechanism involving suppression of phosphatidyl inositol-3-kinase/AKT and p38/ERK MAP kinase signaling and inhibition of RUNX2 phosphorylation and activation (98). More recent studies extended this work by showing that DDR1 up-regulates its own synthesis in response to the stiffness of the matrix environment around VSMCs. This is accomplished by stimulating the nuclear translocation of the Hippo pathway intermediates, YAP and TAZ, to increase *Ddr1* transcription and subsequent mineralization (99). This may explain the known relationship between arterial stiffening and acceleration of vascular calcification (100).

### 7.2. Osteoarthritis

Osteoarthritis (OA), a primary indicator for joint degeneration, is characterized by cartilage degradation, osteophyte formation and joint mineralization (101). OA can occur in fibrocartilage of the temporomandibular joint (TMJ) or in hyaline cartilage of major joints such as the knee. OA in hyaline cartilage generally increases with age. In contrast, TMJ OA has an earlier onset (102, 103). Interactions between chondrocytes and the ECM of hyaline cartilage and fibrocartilage may be key factors for understanding OA pathogenesis in these two tissues. TMJ fibrocartilage extracellular matrix mainly contains type I collagen while type II collagen predominates in hyaline joints (104). Both DDR1 and DDR2 are involved in OA etiology although they may function through different mechanisms. Unlike DDR1, which is broadly but weakly activated by collagens I to IV, DDR2 is strongly activated by types I and III collagen of TMJ fibrocartilage but is less responsive to type II collagen (28). *Ddr2* is expressed at low levels in healthy adult hyaline cartilage joints but is abundant in TMJ fibrocartilage (58). Thus, *Ddr2* is normally expressed at highest levels in an ECM environment that is conducive to its activation. Consistent with its distribution, *Ddr2* is required for normal TMJ formation; global *Ddr2* inactivation disrupts TMJ development beginning in neonates which show an initial delay in condyle mineralization that persists in adults leading to eventual joint degeneration and subchondral bone loss (58). In contrast, knee joints, which are composed of hyaline cartilage, are not affected by *Ddr2* deficiency. *Ddr1* global knockout mice, in contrast, exhibit a spontaneous rapid-onset TMJ OA that is seen by 9 weeks without involvement of other joints (105). The authors of this study proposed that induction of TMJ OA is related to the observation that loss of DDR1 was accompanied by a compensatory up-regulation of DDR2. This is then activated by the type I collagen in TMJ fibrocartilage to induce OA. It is not known if these changes are seen in *Ddr1*-deficient neonates although a separate study reported TMJ abnormalities in mice as young as 4 weeks (50).

DDR2 has also been related to OA in hyaline cartilage joints. In this case, the normally low levels of DDR2 in adults are increased with injuries such as trauma or surgical destabilization of the medial meniscus, which subsequently induce OA (106). In this case, globally *Ddr2*-deficient mice or mice where *Ddr2* is selectively inactivated in articular cartilage are resistant to surgically-induced OA indicating that DDR2 is required for OA induction in this tissue (107, 108). However, overexpressing *Ddr2* in hyaline cartilage does not lead to spontaneous OA formation unless hyaline cartilage ECM is altered by trauma (106, 108). It has been proposed that trauma-induced damage to the ECM may disrupt the pericellular matrix around chondrocytes and allow them to interact with type II collagen fibrils resulting in DDR2 activation and OA (107).

### 7.3. Heterotopic ossification

Heterotopic ossification (HO) is a debilitating condition that occurs after many traumatic injuries. In HO, PDGFRα+ connective tissue cells present in soft tissue adjacent to the injury site change their differentiation trajectory to form ectopic cartilage and bone (109). *Ddr2* has been recently shown to play a role in the pathogenesis of HO (93). Using single cell RNA sequencing, *Ddr2* was discovered to be highly expressed by PDGFRα+ cells, that form the major cell lineages involved in HO formation. In HO, both DDR2 and phospho-DDR2, a marker of active DDR2, were shown to be significantly upregulated in PDGFRα+ cells within the tendon, peritendon, and soft tissue areas surrounding the HO site. Interestingly, DDR2 mediates HO formation after injury, as both *Ddr2^slie/slie^* mice (global knockout) and tamoxifen-treated *Pdgfa-Cre^ER^*;*Ddr2^fl/fl^* mice (conditional knockout in progenitor cells) display significant reductions in *Sox9* expressing chondrocytes, safranin O-labeled cells and reductions in ectopic bone formation due to extracellular matrix disorganization and FAK/YAP/TAZ dysregulation (described in Section 5). This study highlights how extracellular matrix alignment can have profound effects on HO progression and how DDR2 is an important regulator of this process.

## 8. Metabolic effects of Ddr2 deficiency and relationship to bone metabolism

In addition to inhibiting skeletal growth, global *Ddr2* deficiency also affects metabolism. For example, *Ddr2^slie/slie^* mice have elevated blood glucose levels, reduced body fat and increased lean body mass (75), elevated levels of circulating adiponectin and decreased serum leptin (65). It is not known if there is a relationship between these metabolic changes and the bone phenotype of these mice. However, as discussed in Sections 4.1, 4.3, the decrease in bone mass in *Ddr2^slie/slie^* mice is paralleled by an increase in marrow fat, a change that may be related to the reduced ERK/MAPK activity in mutant mice. The consequences of this reduced MAPK activity would include suppression of RUNX2 and PPARγ phosphorylation, decreased osteoblast and increased marrow adipocyte gene expression and differentiation. Since marrow adipocytes are a major source of serum adiponectin (110), the increase in marrow adipocytes in *Ddr2^slie/slie^* mice may explain the observed increase in serum adiponectin. However, specific knockout of the Adipoq gene in marrow adipocytes using a recently described double recombination strategy (111) would be necessary to definitively test this hypothesis.

Interestingly, *Ddr2* is expressed in adipocytes. Early studies suggested possible direct effects of DDR2 on these cells such as suppression of insulin stimulated tyrosine phosphorylation of the insulin receptor in the 3T3-L1 adipocyte cell line (112). More recently, direct effects of DDR2 on adipocytes *in vivo* were examined using *Adipo^Cre^*;*Ddr2^fl/fl^* mice, where *Ddr2* is inactivated in peripheral as well as marrow fat (113). In this study, mutant mice were protected from high fat diet-induced weight gain, a response that was attributed to decreased adipocyte size. Significantly, these animals also had a high bone mass phenotype accompanied by increases in both bone formation rate and resorption. These changes were explained by a DDR2-specific repression of adenylate cyclase 5 (Adcy5) in adipocytes removed in mutant mice leading to increased cAMP production and lipolysis in marrow adipocytes. The released fatty acids in the marrow cavity then promote increased oxidative metabolism in osteoblast leading to increased osteoblast and osteoclast activity. Therefore, by modulating lipolysis in adipocytes, DDR2 can indirectly control bone formation. This mechanism may complement the more direct effects of DDR2 on skeletal progenitor cells described in Section 4.3.2.

## 9. Summary and future perspectives

The study of DDR functions in bone is a relatively new research area and many questions remain about what these collagen receptors do and how they do it. As shown in this review, both DDR1 and DDR2 have functions in mineralized tissues with DDR2 perhaps having a greater role under physiological conditions. However, clear functions for DDR1 are also seen, particularly in pathological conditions such as vascular calcification.

Although tissue distribution studies, particularly for DDR1, are incomplete, the original conclusion that DDR1 functions in epithelia while DDR2 is in connective tissues may need revision, particularly for DDR1, which has clear functions in connective tissues like cartilage and bone. More detailed DDR1 localization and lineage tracing studies will be required to more fully understand where this collagen receptor functions. The observation that DDR2 is present in GLI1-positive skeletal progenitor cells of cranial sutures and, possibly, cartilage where it controls cell proliferation and differentiation to chondrocytes and osteoblasts is of particular interest. These studies suggest that DDR2, together with collagen binding integrins, allows certain classes of skeletal progenitor/stem cells to sense their ECM environment and modulate their differentiation state according to ECM stiffness and mechanical loads. As the more ancient of the two collagen receptors, the DDRs were likely complemented by the newly emerging collagen-binding integrins when the vertebrate skeleton first evolved so that these two receptors now work in concert. Another intriguing area is the possible function of DDR2 in osteoimmunology where it may modulate activities of various myleloid lineages to control inflammation and bone resorption.

**Figure 2:**
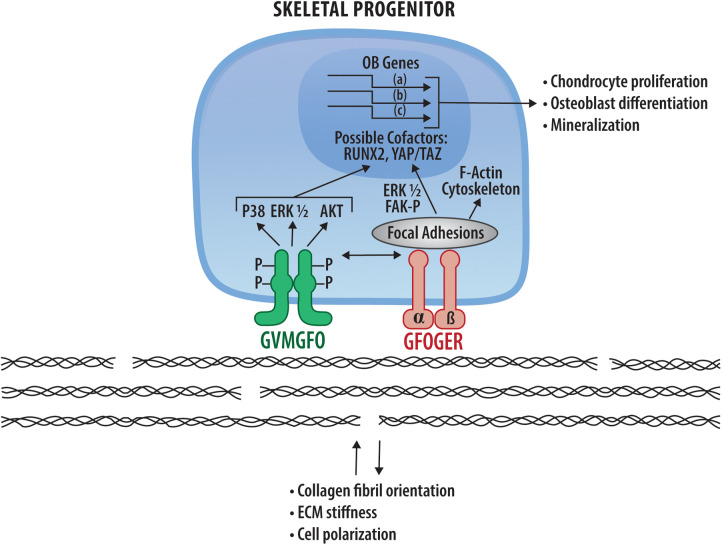
DDR2 signaling mechanisms in bone and potential interactions with collagen-binding integrins. DDR2 (green) and integrins (pink) bind and are activated by the specific sequences in fibrillar collagens indicated. Downstream signals arising from DDR2 and integrin activation are indicated as are nuclear changes in RUNX2 and HIPPO pathway intermediates, YAP and TAZ, resulting in transcriptional changes necessary for chondrocyte proliferation, osteoblast differentiation and mineralization (see Sections 4.4). Also shown are DDR-dependent changes in collagen matrix organization and cell polarity described in Section 6.

Although conditional knockout studies showed that DDR2 functions in skeletal progenitor cells and chondrocytes, little is known about its actual mechanism of action in these tissues. Current, albeit incomplete, knowledge in this area is summarized in Figure 2. Some of its activities may be explained by modulation of MAP kinases which subsequently control osteogenic and adipogenic transcription factors through phosphorylation. However, this is likely only part of the story. The dramatic effects of DDR2 on collagen fibril orientation, matrix stiffness and cell polarity may also be an important part of an overall mechanism that still needs to be discerned. By modulating matrix stiffness-associated pathways including the Hippo pathway, DDR2 and integrins may work together to control stiffness-associated nuclear changes and transcription. These matrix signals may also modify the response of cells to soluble signals coming from growth factors or morphogens. All these topics are clearly fruitful areas for future investigations.

Recent discoveries on DDR function may also have important implications for the treatment of disease. For example, the demonstrated role of DDR1 in vascular calcification and of DDR2 in osteoarthritis and heterotopic ossification suggest that specific DDR2 inhibitors already under development could be used to treat these disorders (114). Also, the recent discovery that DDR2 is required for skeletal regeneration may open new directions for therapy through the development of either DDR-activating tissue engineering scaffolds or other treatments that modify DDR activity.

Clearly, the study of DDRs in bone will continue to be a growing area of musculoskeletal research that holds much promise for exciting future discoveries.
